# The Prevalence of Variations in the Morphology of Renal Arteries in the North Indian Population

**DOI:** 10.7759/cureus.115056

**Published:** 2026-08-23

**Authors:** Rabiya Bashri, Rajani Singh

**Affiliations:** 1 Anatomy, Graphic Era Institute of Medical Sciences, Dehradun, IND

**Keywords:** aberrant artery, kidney, polar artery, renal artery, variation

## Abstract

Background and objective

The kidneys occur in pairs, and each kidney is supplied by a single artery originating from the abdominal aorta. However, unusually, these arteries supplying the kidneys may originate from sources other than the abdominal aorta and may also be double or triple in number. In addition, the arteries supplying the kidneys through the hilum may also enter the kidneys through their poles. Such arteries are known as polar arteries. The aforementioned variations show ethnic differences. There is a scarcity of literature describing the variations in these arteries in the North Indian population. Hence, this study aimed to highlight the variations in the renal arteries (RAs) in the North Indian population.

Methods

The study was conducted in the Department of Anatomy using 15 formalin-fixed cadavers. The abdomen was opened, the kidneys and RAs were exposed, and the variations were recorded, photographed, and interpreted.

Results

The prevalence of a single RA unilaterally was 66.7% (20/30), while that of a single RA bilaterally was 46.7%. The incidence of double RAs unilaterally was 26.7% (8/30), while that bilaterally was 6.7% (1/15). Polar arteries were observed in two specimens: one on the left side, constituting 6.7% (1/15), and the other on the right side, arising from the abdominal aorta as a common trunk with the RA. All arteries originated from the abdominal aorta.

Conclusions

Knowledge of RA variations will be of immense use to urologists in the diagnosis and management of kidney diseases in the North Indian population.

## Introduction

The kidneys are a pair of excretory organs located on the posterior abdominal wall and remove waste products from the body. Each of these kidneys is supplied by a single renal artery (RA). These renal arteries originate from the abdominal aorta. The right RA is longer than the left RA. The right RA also arises cranial to the left RA. This is the classical anatomy taught to students. Moreover, these RAs, at the renal hilum, divide into two branches. Both these branches give rise to segmental arteries, which supply the segments of the kidneys [1]. However, the RAs are subject to variations in terms of their number, course, and branching pattern. The additional branches that reach the hilum to irrigate the kidneys are referred to as accessory or supernumerary RAs. On the other hand, the RAs that do not enter the kidney through the hilum, but instead enter the kidneys through the poles, are known as aberrant renal arteries or polar arteries [1].

Literature on these arterial variations in specific Indian populations remains scarce. These variations of renal arteries exhibit ethnic differences. Therefore, information regarding the morphological variations of RAs in the North Indian population will help nephrologists and vascular surgeons in planning and managing renal ailments in North Indians, thereby reducing morbidity and mortality. Hence, the study is of importance. The objective of the study is to highlight the different variations of renal arteries in the North Indian population. Knowledge of variations in renal arteries will be of immense use to urologists worldwide. The study aims to explore the variations in the morphology of RAs in the North Indian population.

## Materials and methods

The study was conducted in the Department of Anatomy, Graphic Era Institute of Medical Sciences (GEIMS), Dehradun, India, using 15 formalin-fixed adult cadavers aged 55-83 years. The cadavers comprised nine male and six female cadavers, with an average age of 69 years. The abdomen was dissected using the standard dissection technique described in Cunningham’s Dissecting Manual. The abdominal aorta, kidneys, and RAs were exposed. The RAs were traced from their origins to the kidneys. The origin and number of RAs, their entry into the renal hilum or poles, and their division either in the pre-hilar region or at the hilum were noted and photographed. The data were collected and entered into an Excel spreadsheet, and the prevalence was calculated and interpreted.

Only adult cadavers without any evidence of renal disease were included in the study. Pediatric cadavers were excluded from the study. The study was conducted to identify and document remarkable variations in the renal arteries. The literature was reviewed and compared with previously published studies. The associated clinical implications were also correlated with the variant morphology of the RAs. The study was approved by the Institutional Review Board vide approval No. GEIMS/IRB/2026/1, Cycle 7.

## Results

**Figure 1:**
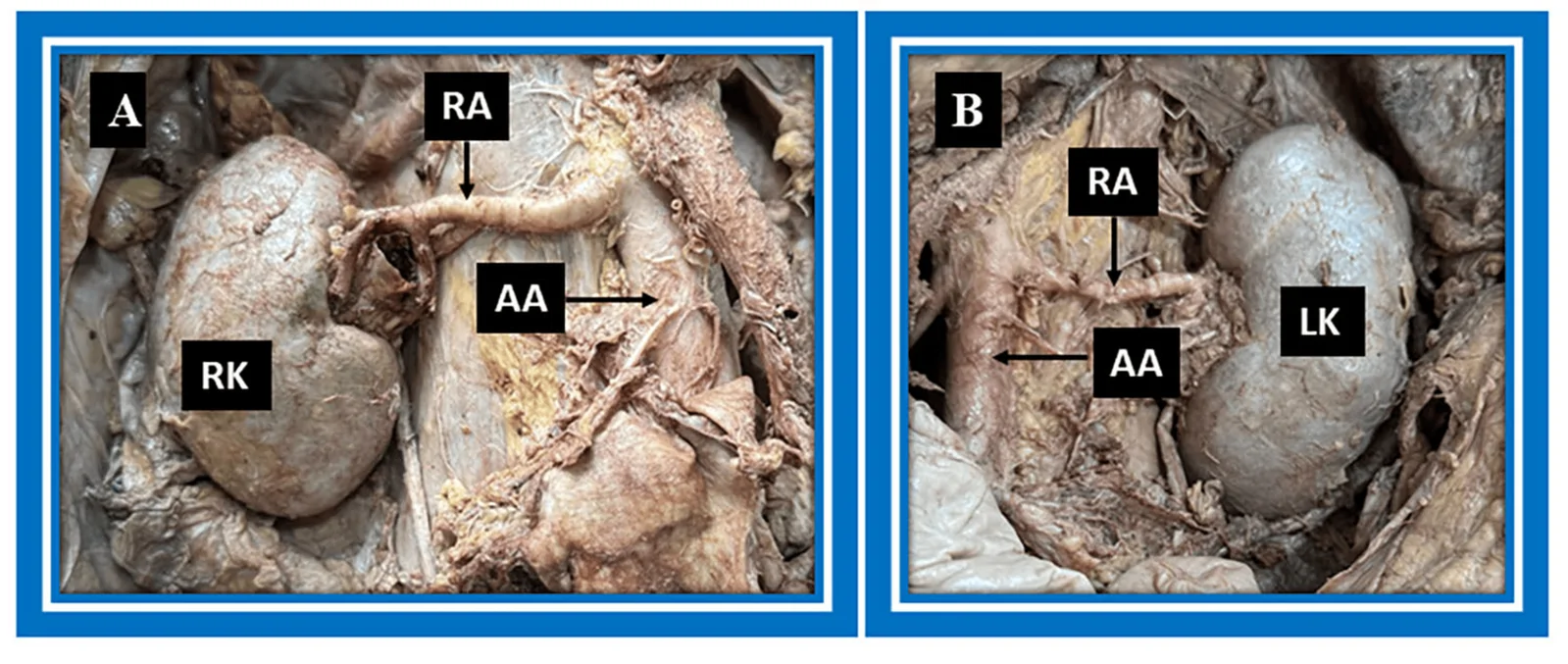
Photographs of single renal artery A: single renal artery on the right side; B: single renal artery on the left side RA: renal artery; RK: right kidney; AA: abdominal aorta; LK: left kidney

A total of 30 kidneys (15 pairs) were examined for variations in the morphology of the RAs supplying the kidneys. A single RA was observed in 12 right kidneys (Figure 1A), constituting 80% (12/15), and in eight left kidneys (Figure 1B), with an incidence of 53.3% (8/15).

**Figure 2:**
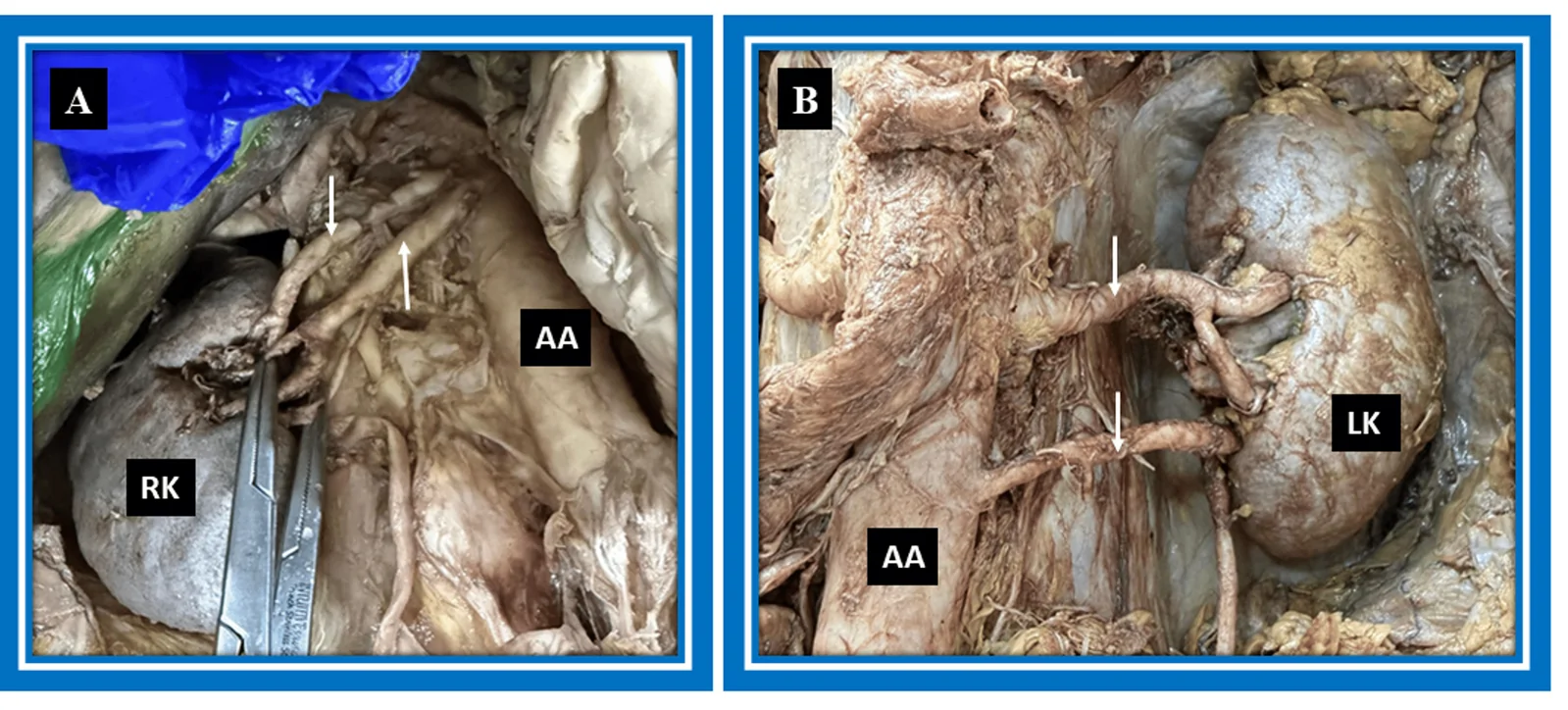
Photographs of double renal artery A: double renal artery on the right side; B: double renal artery on the left side RK: right kidney; AA: abdominal aorta; LK: left kidney; white arrows point to the renal arteries

The prevalence of a single renal artery unilaterally was 66.7% (20/30). A single renal artery bilaterally was detected in seven specimens (7/15), constituting 46.7%. Accessory renal arteries were observed unilaterally in eight kidneys, accounting for 26.7% (8/30), with two occurring in the right kidneys (2/30) and six in the left kidneys (6/30), constituting 13.3% and 40% on the right and left sides, respectively. The incidence of double renal arteries (Figures 2A, 2B) unilaterally was 26.7%, while the incidence bilaterally was 6.7% (1/15), observed in one cadaver. Triple renal arteries were not observed in any of the specimens.

**Figure 3:**
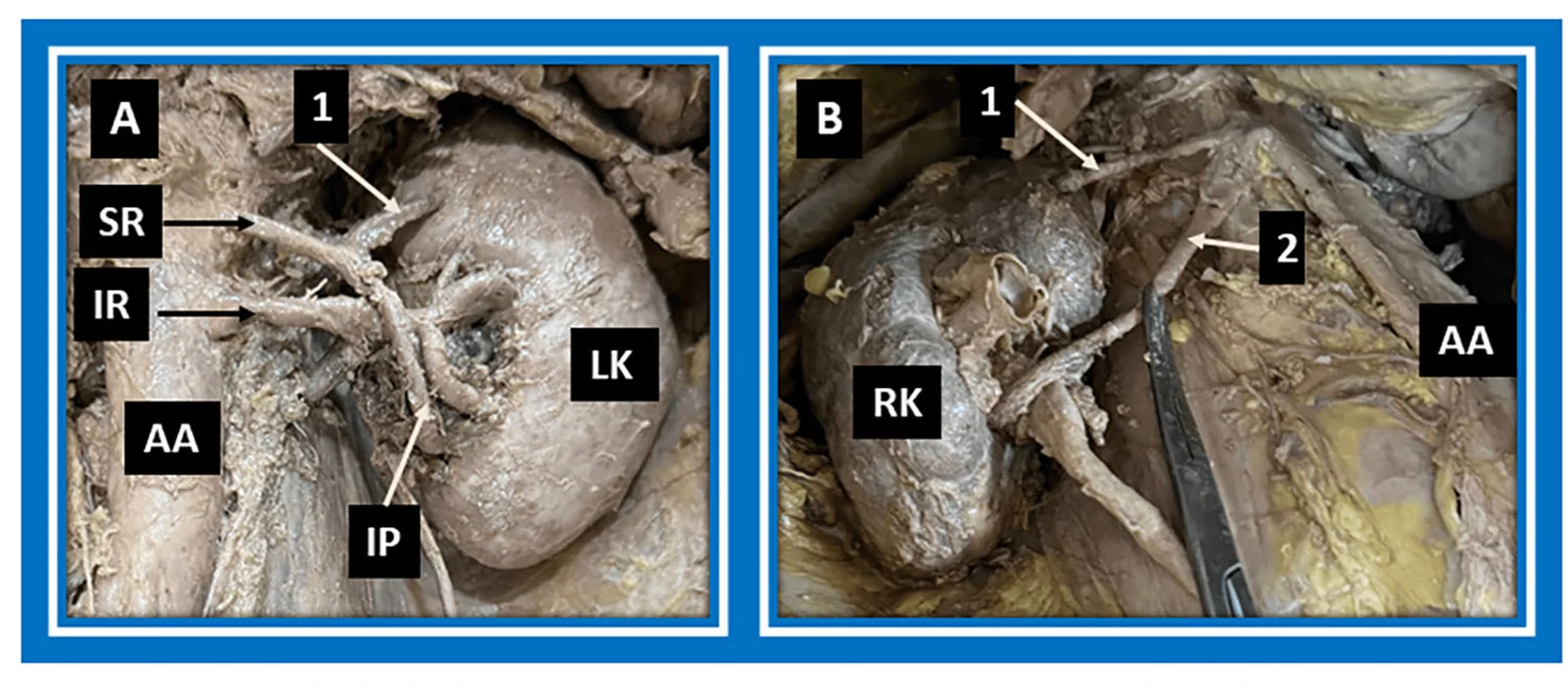
Photographs of aberrant renal arteries or polar arteries A: superior and inferior polar arteries from the left renal artery; B: superior renal artery in common with the renal artery from the abdominal aorta on the right side RK: right kidney; AA: abdominal aorta; LK: left kidney; SR: superior renal artery; IR: inferior renal artery; IP: inferior polar artery; 1: superior polar artery; 2: renal artery

In all cases, the single and accessory RAs originated from the abdominal aorta. Aberrant RAs in the form of polar arteries were observed in two specimens: one arising from the left RA (Figure 3A), constituting 6.7% (1/15), and the other arising as a common trunk with the right RA (Figure 3B) from the abdominal aorta.

**Figure 4:**
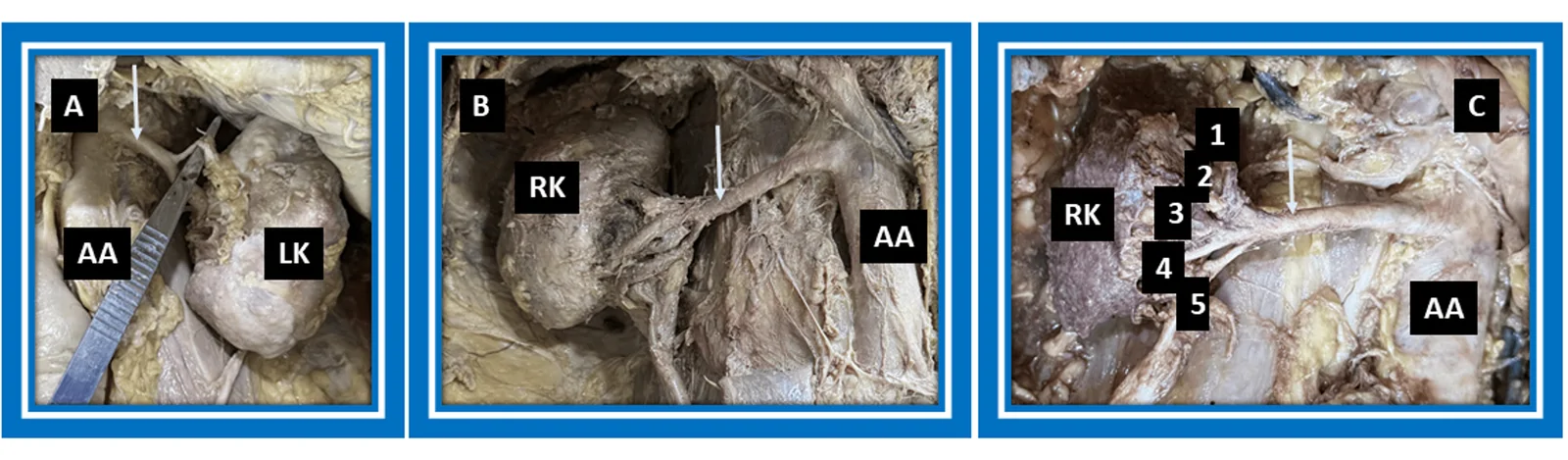
Photographs of pre-hilar division of renal artery A: pre-hilar division of the renal artery with two sub-branches on the left side; B: pre-hilar division of the renal artery with three sub-branches on the right side; C: pre-hilar division of the renal artery with five sub-branches on the right side RK: right kidney; AA: abdominal aorta; LK: left kidney; 1, 2, 3, 4, 5: sub-branches of the renal artery on the right side; white arrows point to the renal arteries

Pre-hilar division was observed unilaterally in five kidneys (5/30), constituting 16.7%, with 20% (3/15) observed on the right side and 13.3% (2/15) on the left side. Bilateral pre-hilar division was detected in six cadavers (6/15), constituting 40%. Pre-hilar division into two to three branches (Figures 4A, 4B) was observed in most cases. However, pre-hilar division into five branches (Figure 4C) was observed in the right kidney, representing a rare finding.

**Figure 5:**
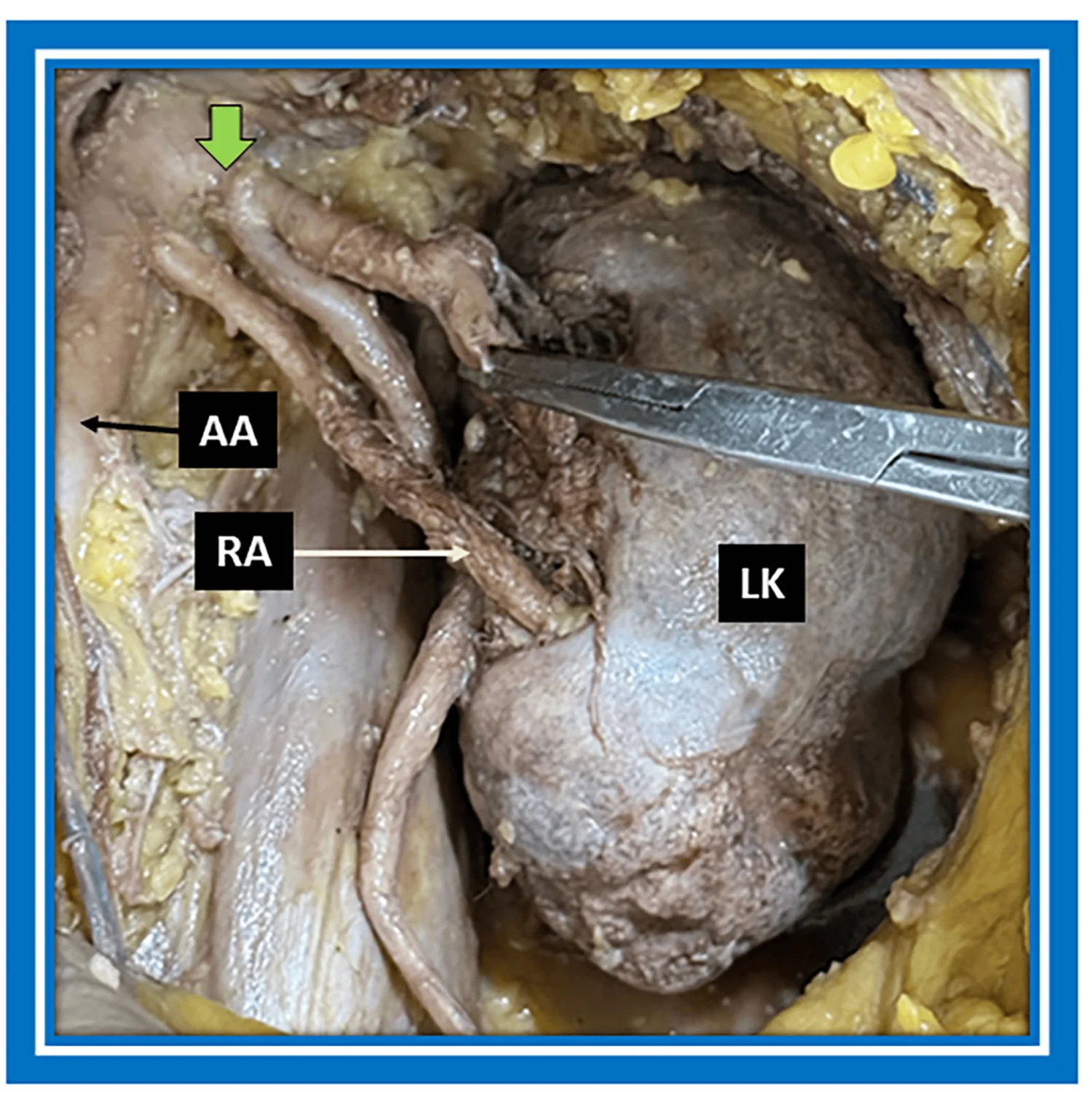
Pre-hilar division of the common trunk from the abdominal aorta occurring 0.3 cm after originating from the abdominal aorta AA: abdominal aorta; LK: left kidney; RA: renal artery; green arrow: common trunk from aorta

One interesting finding observed in the current study was that the pre-hilar division in two left kidneys occurred 0.3 cm after originating from the abdominal aorta (Figure 5).

## Discussion

Conventionally, the RAs originate from the abdominal aorta but may also arise from sources other than the abdominal aorta. The incidence of a single RA originating from the abdominal aorta has been reported to range from 63%-80% [2-3]. In the present study, the incidence of a single RA originating from the abdominal aorta, both unilaterally and bilaterally, was 66.7% (20/30) and 46.7% (7/15), respectively, which falls within the reported range. The RAs present in addition to the main RA that supply the kidneys by entering through the hilum are referred to as accessory, multiple, or supernumerary RAs, whereas additional RAs that do not enter the kidneys through the hilum but instead reach the kidneys through the poles are referred to as aberrant or polar arteries [3].

The most frequent arterial variation of the kidneys is the presence of an additional RA, with an incidence of 30% [4]. According to other authors, the incidence of multiple RAs ranges from 2% to 36% [5], with the highest incidence of 36% reported recently by Arasu et al. [6]. The incidence of more than one RA, both unilaterally and bilaterally, in the present study was 26.7% (8/30) and 6.7% (1/15), respectively, which aligns with the findings of the aforementioned studies. However, in this study, the incidence of accessory RAs on the right and left sides was 13.3% (2/15) and 40% (6/15), respectively.

The incidence of double and triple RAs reported in the literature has been observed to be 20% [7] and 25-30%, respectively [7-9]. In this study, the incidence of double RAs was 26.7% unilaterally and 6.7% bilaterally, which was higher than that reported in the literature [7]. However, triple RAs were not observed in any of the specimens. Accessory RAs may arise from the aorta or the main RA [10] or from other vessels, including the thoracic aorta, celiac artery, inferior phrenic artery, gonadal artery, superior mesenteric artery, inferior mesenteric artery, common iliac artery, external iliac artery, and internal iliac artery [4,11].

In the current study, all accessory RAs arose from the abdominal aorta. Aberrant RAs entering the kidneys at the poles are classified into superior and inferior polar arteries [5]. The reported incidence of superior and inferior polar arteries ranges between 3.3% and 9.3% and 5% and 29.6%, respectively [5]. However, in the same year, another study reported an incidence of 78% for superior polar RAs and 28% for inferior polar arteries [10]. In this study, aberrant RAs were found in only two kidneys. In one case, the artery arose from the main RA on the left side, constituting 6.7% (1/15), while in the other case, the superior polar artery arose as a common trunk with the RA from the abdominal aorta. These incidences of aberrant arteries are well within the range reported by other authors, such as Kaushik et al. [5], but are considerably lower than those reported in another study [10]. Hence, other variations are also important to consider when evaluating the origins and courses of arteries arising from the RA and abdominal aorta.

Pre-hilar division of renal artery

Under standard anatomical conditions, the RAs divide into two branches at the hilum of each kidney. However, bifurcation of the RAs before reaching the hilum, known as pre-hilar division of the renal artery, has been reported in the literature with an incidence ranging from 8.12% to 21% [10,12]. Another study conducted in 2024 reported an incidence of pre-hilar division of the renal artery of 38.3% [5]. In the present cadaveric study, pre-hilar division of the renal artery was observed in 16.7% (5/30) and 40% (6/15) of the kidneys unilaterally and bilaterally, respectively, which is consistent with the findings of the aforementioned studies.

Developmental cause of the formation of accessory renal arteries, including their pre-hilar division

In 1912, Felix proposed a theory explaining the formation of accessory RAs, which is still accepted today, as quoted by Manoharan et al. [11]. According to this theory, nine pairs of mesonephric arteries originate from the aorta and supply blood to the progressively developing kidneys, suprarenal glands, and gonads. The definitive RAs develop from the third and fourth pairs of mesonephric arteries, while the remaining mesonephric arteries regress. When the mesonephric arteries that normally degenerate persist, they form accessory RAs [13].

In addition to the mesonephric arteries, the fetal kidneys are also supplied by nearby arteries, such as the common iliac arteries and the aorta. Sometimes, these arteries fail to involute and persist, explaining the origin of RAs from sources other than the aorta [14]. During renal development, various factors, such as glial cell-derived neurotrophic factor and hepatocyte growth factor present in the arterial mesenchyme, interact with factors present in the metanephric mesenchyme [14]. However, if this communication between the aforementioned factors does not occur at the appropriate time, it may result in pre-hilar bifurcation of the RA [14].

Clinical significance of variant morphology of renal arteries

A proper understanding of accessory RAs is essential for reducing the risk of hemorrhagic episodes during suture placement, interpreting angiograms, and performing kidney transplantation [7]. Information regarding the presence of accessory RAs provides valuable insights for donor selection during kidney transplantation, renal artery embolization, arterial reconstruction procedures, and the management of aneurysms involving the descending aorta [7]. With the increasing use of laparoscopic procedures, a detailed understanding of accessory renal arteries has become essential. In addition, understanding these variations is of immense value in preventing ischemic changes in renal segments during partial renal resection [15].

Aberrant RAs in the form of polar arteries that course anterior to the renal pelvis may compress the renal pelvis, resulting in complications such as hydronephrosis and urinary tract infections [11]. Polar arteries may also be inadvertently damaged during dissection around the poles of the kidneys [11]. Hence, knowledge of these aberrant arteries is of paramount importance to urologists. Moreover, information regarding pre-hilar division of RAs is essential for ensuring adequate arterial anastomosis during kidney transplantation, as the length of the renal artery should be greater than 2 cm for these procedures [16]. However, in the present study, pre-hilar division occurred 0.3 cm from the origin of the RA from the aorta. This finding warrants the attention of urologists and vascular surgeons during kidney transplantation.

## Conclusions

All the RAs in the present study arose from the abdominal aorta. The incidence of double RAs varied between the left and right kidneys, consistent with previously reported studies. The incidences of double RAs observed unilaterally and bilaterally in the present study were also within the ranges reported in the literature. The incidence of pre-hilar division of the RAs in the present study was higher than that reported in the literature. In two kidneys, the length of the RAs before pre-hilar division was only 0.3 cm, whereas the required length of the RA for kidney transplantation is more than 2 cm. As variations in the morphology of renal arteries are subject to ethnic differences and vary among populations, the information provided will be of immense use to urologists and vascular surgeons in the diagnosis and management of kidney diseases in the North Indian population. The study included only 15 pairs of kidneys, which is a limitation. Hence, further studies with larger sample sizes are recommended.
